# Supplementary material for: The Mutational Landscape of Acute Myeloid Leukaemia Predicts Responses and Outcomes in Elderly Patients from the PETHEMA-FLUGAZA Phase 3 Clinical Trial
Source: Cancers (Basel). 2021 May 18;13(10):2458. doi: 10.3390/cancers13102458 (PMC8158477; doi:10.3390/cancers13102458)
Supplement: Supplementary file 1 [file cancers-13-02458-s001.zip › cancers-1203671-supplementary/cancers-1203671-supplementary.pdf]

cancers

MDPI

Supplementary Materials

The Mutational Landscape of Acute Myeloid Leukaemia Predicts Responses and Outcomes in Elderly Patients from the PETHEMA-FLUGAZA Phase 3 Clinical Trial

Rosa Ayala, Inmaculada Rapado, Esther Onecha, David Martínez-Cuadrón, Gonzalo Carreño-Tarragona, Juan Miguel Bergua, Susana Vives, Jesus Lorenzo Algarra, Mar Tormo, Pilar Martinez[0], Josefina Serrano[1], Pilar Herrera, Fernando Ramos, Olga Salamero, Esperanza Lavilla, Cristina Gil, Jose Luis Lopez-Lorenzo, María Belén Vidriales, Jorge Labrador, Jose Francisco Falantes, María José Sayas, Bruno Paiva, Eva Barragán, Felipe Prosper, Miguel Ángel Sanz, Joaquín Martínez-López and Pau Montesinos and on behalf of the Programa para el Estudio de la Terapeutica en Hemopatias Malignas (PETHEMA) Cooperative Study Group

FLUGAZA Clinical Trial (PETHEMA)

Patients

Two-hundred and eighty-three patients aged over 65 with newly-diagnosed acute myeloid leukemia (AML; excluding those with acute promyelocytic leukemia according to World Health Organization criteria 1) and with an Eastern Cooperative Oncology Group PS (ECOG PS) <4. were enrolled in the PETHEMA phase 3 FLUGAZA clinical trial (NCT02319135). Exclusion criteria included prior treatment with hypomethylating agents or standard chemotherapy for AML secondary to myelodysplastic syndrome or myeloproliferative neoplasms, inadequate renal and hepatic function unless attributable to AML, and presence of other major coexisting illnesses (except *in situ* carcinoma or concomitant malignancy in complete remission [CR] for more than one year). All patients provided written informed consent. The trial was approved by appropriate institutional review boards or ethics committees at all sites before initiation and was conducted according to the tenets of the Declaration of Helsinki and the Harmonization E6 Guidelines for Good Clinical Practice.

Treatment.

Patients were randomized 1:1 to receive open-label treatment with azacytidine or with low-dose cytarabine plus fludarabine (FLUGA). The induction phase consisted of three cycles. Patients in the experimental arm received subcutaneous (s.c.) azacytidine in standard doses (75 mg/m^2) on days 1 to 7 of each cycle (5-2-2 administration was allowed). Concomitant oral hydroxycarbamide (0.5–1 g every 8 hours) was administered in addition to azacytidine when white blood cell (WBC) counts were between 15-50 × 10^9/L until leukocytes decreased to <15 × 10^9/L. Patients assigned to the azacytidine arm with WBC >50 × 10^9/L received the FLUGA scheme instead of azacytidine in cycle 1. Patients in the FLUGA arm received cytarabine (75 mg/m^2) by s.c. administration or 6-hour intravenous (i.v.) perfusion when they were outpatients or hospitalized, respectively, together with fludarabina, either oral at 40 mg/m^2 if an outpatient or i.v. at 25 mg/m^2 if hospitalized. on days 2 to 6 (days 2 to 5 when they were ≥75 years old). Patients in this arm also received s.c. filgrastim (granulocyte-colony stimulating factor; 5 μg/kg) on days 1-3 except when WBC were >25 × 10^9/L. Cycles were repeated every 28 days. Criteria to receive treatment as an inpatient included WBC >25 x10^9/L. high risk of tumor lysis syndrome coagulopathy or other serious uncontrolled complication. Patients in CR. complete remission with incomplete blood count recovery (CRi), partial remission (PR), hematology improvement or stable disease after the induction phase continued with the consolidation phase., which consisted of six cycles that also lasted for 28 days. In the experimental arm, the dose route and days of administration of azacytidine were the same as those used in the induction

phase. In the FLUGA arm, daily doses and routes of administration of FLUGA were the same as those used during induction, but drugs were given only on days 1 and 2 of every cycle (mini-FLUGA). All patients could receive supportive care (transfusions. antimicrobial and antifungal agents) as per institutional standard practice. An allogeneic hematopoietic stem cell transplant was not indicated as part of the front-line strategy in this clinical trial.

At the end of the ninth cycle, patients in CR/CRi had bone marrow aspirates for assessment of measurable residual disease (MRD). Those with MRD levels ≥0.01% continued treatment (azacytidine or mini-FLUGA) until relapse or progressive disease was documented. Patients whose MRD levels were <0.01% suspended treatment and entered the follow-up phase.

Supplemental Methods.

Mutational profile workflow and filtering and classification of variants was performed as previously published (Onecha E. Haematologica 2020)

Patients were evaluated for mutations in *NPM1* and *FLT3* by methods other than next generation sequencing (NGS) (PCR and GeneScan. respectively). We used a separate script and detected the *FLT3*-ITD mutation by NGS. Although this panel was not designed to detect large InDels. we only missed 5 cases in the series who were positive using GeneScan analysis and these cases were considered as positive *FLT3*-ITD.

Custom NGS panel which included 43 genes implicated in myeloid pathology outlined below.

GENE	CHR	START	END	COVERAGE (%)
ASXL1	20	30954122	31025231	98.59
BCOR	X	39911228	39937243	100
BCORL1	X	129139130	129190192	97.03
CALR	19	13049460	13054786	100
CBL	11	119077080	119170509	96.95
CEBPA	19	33792147	33793455	97.57
CSF3R	1	36931652	36945167	100
DNMT3A	2	25457047	25536929	96.81
EGLN1	1	231502062	231557733	89.26
EPAS1	2	46525036	46611847	95.38
EPOR	19	11488599	11495008	93.96
ETV6	12	11802967	12044078	100
EZH2	7	148504657	148544423	100
FLT3	13	28578144	28644795	98.36
IDH1	2	209101731	209116356	100
IDH2	15	90627367	90635017	84.96
JAK2	9	5021946	5126835	100
KDM6A	X	44732709	44970753	99.01
KIT	4	55524176	55604767	100
KMT2A	11	118307241	118393002	97.7
KRAS	12	25362705	25398385	100
MPL	1	43803488	43818462	99.26
NF1	17	29422227	29701206	99.66
NPM1	5	170814868	170837656	95.27
NRAS	1	115251106	115258821	100
PHF6	X	133511597	133559416	89.4
PRPF40B	12	50017325	50038043	99.18
RAD21	8	117859710	117878977	100
RUNX1	21	36164287	36421263	98.42
SETBP1	18	42281301	42643812	100
SF3A1	22	30730553	30752861	100
SF3B1	2	198256921	198299857	98.87

SH2B3	12	111855923	111886159	88.57
SMC1A	X	53406965	53449648	98.28
SRSF2	17	74732208	74733436	100
STAG2	X	123156407	123234509	100
TET2	4	106154899	106197684	100
THPO	3	184090090	184096202	100
TP53	17	7572852	7579966	94.5
U2AF1	21	44513191	44527685	98.86
VHL	3	10183360	10191667	96.58
WT1	11	32410545	32456973	91.46
ZRSR2	X	15808512	15841397	100

Table S1. Patient characteristics.

**Variables**		**NGS study (*N* = 207)**
Age at diagnosis	Years. median (range)	75 (65–90)
Blasts at diagnosis	%. median	53
WBC at diagnosis	$\times 10^{-9}$/L. median (range)	6.7 (0.56–235)
Dyserythropoiesis	n. cases. %	92 (44%)
Dysmyelopoiesis	n. cases. %	80 (39%)
Dysthrombopoiesis	n. cases. %	54 (26%)
AML origin	de novo	116 (56%)
	AML secondary MDS	92 (44%)
	AML secondary treatment	16 (8%)
FAB classification	M0/M1/M2/M4/M5/M6/M7/NOS	32/ 36/ 35/ 1/ 43/ 24/ 10/ 19
Cytogenetics	Abnormal karyotype	97 (47%)
Cytogenetics Risk Group (ELN criteria)	Low	16
	Intermediate	84
	High	97
WHO classification	AML with certain recurrent genetic abnormalities	18
	AML wth myelodysplasic-related changes	92
	AML related to previous chemotherapy or radiation	16
	AML NOS	80
Induction treatment	AZA	96
	LDAC (FLUGA)	111
3rd cycle response	CR	54 (26%)
	PR	21 (10%)
Progression cases		131 (63%)
Death cases		167(81%)

Table represents the clinical data of patients included in the FLUGAZA clinical trial with an NGS gene panel study at diagnosis. WBC = white blood cells. AML = acute myeloid leukemia. AZA = azacitidine. ELN = European leukemiaNet (2017). MDS = myelodysplastic syndromes. FAB= French-American-British. NOS = not otherwise specified. LDAC (FLUGA) = low-dose cytabrine plus fludarabine. CR = complete remission and PR = partial remission. Clinical data were collected from the FLUGAZA trial (NCT02319135).

Table S2. Comparison of clinical and biological characteristics of the 207 patients who were included in this study and 78 not included The number of partial responses in the 78 cases without molecular evaluation is in agreement with that obtained in the population included in this study in function of treatment received.

Variable		**AZA-Arm (*N* = 96)**	**FLUGA-Arm (*N* = 111)**	**Patients Excluded (*N* = 78) AZA-arm =47 FLUGA-arm = 32**	***p*-Value**
Age at diagnosis	Years. media	75	76	76	p = NS
Blasts at diagnosis	%. media	55	53	47	p = NS
WBC at diagnosis	x10^{-9}/L. media	22	21	19	p = NS
Dyserythropoiesis	n cases	45	47	28	p = NS
Dysmyelopoiesis	n cases	38	42	32	p = NS
Dysthrombopoiesis	n cases	23	31	18	p = NS
AML origin	de novo	*44*	55	47	p = NS
	AML secondary MDS	47	45	30	p = NS
	AML secondary	5	11	1	p = NS
FAB classification	M0/M1/M2/M4/M5/ M6/M7/NOS	16/15/13/1/21/ 12/5/9	16/21/22/0/22/ 12/5/10	6/11/13/10/1 0/4/16	p = NS
Cytogenetics	Abnormal Karyotype/Normal Karyotype	46/ 38	51/26	37/ 29	p = NS
Cytogenetics Risk Group	Low-Intermedite	63	68	50	p = NS
	High Risk	30	35	23	
WHO classification	AML with certain genetic abnormalities	5	13	2	p = NS
	AML with myelodisplasic-related changes	47	45	30	
	AML related to chemotherapy or radiation previous	5	11	1	
	AML NOS	38	42	46	
Follow-up time	Months. median	9.6	7.8		p = NS
3rd cycle response	CR	23	31	15	p = NS
	Cri	14	18	26	p = NS
	PR	16	5	11	p = 0.004
Final response	CR	26	31	16	p = NS
Death cases		73	94	55	p = NS

Table S3. Logistic regression analysis. Response defined as CR after 3rd cycle in the global series in function of characteristics of patients with AML on the treatment arm. Responder patients were defined as patients who achieved CR or CRi after 3rd cycle. AZA = azacitidine. FLUGA = fludarabine plus low-dose cytarabine (LDAC). OR = odds ratio. CI = confidence interval. Cytogenetic Risk Group (Low-Intermediate/High-Risk. classification ELN 2017). The multivariate analyses showed that no variable was associated with achieving a CR after bonferroni adjustment.

Parameter	*p* Value	OR	95% CI for OR	
			Lower	Upper
AZA vs FLUGA-arm	0.438	1.398	0.599	3.265
Median age. years	**0.037**	**0.916**	**0.843**	**0.995**
Cytogenetic (Low-Intermediate / High Risk)	0.626	1.320	0.433	4.029
Mutated *ASXL1* (Yes/No)	0.779	0.837	0.242	2.897
Mutated *BCOR* (Yes/ No)	0.525	0.484	0.051	4.553
Mutated *BCORL1* (Yes/ No)	0.758	1.369	0.186	10.10
Mutated CALR (Yes/ No)	0.999	0	0	.
Mutated *CBL* (Yes/ No)	0.996	1.008	0.048	21.208
Mutated *CEBPA* by NGS (Yes/ No)	0.615	0.606	0.086	4.263
Mutated *DNMT3A* (Yes/ No)	0.422	0.614	0.186	2.023
Mutated *EPAS1* (Yes/ No)	0.268	0.201	0.012	3.431
Mutated *EPOR* (Yes/ No)	0.294	6.317	0.202	197.69
Mutated *ETV6* (Yes/ No)	0.175	0.211	0.022	1.999
Mutated *EZH2* (Yes/ No)	0.130	4.425	0.646	30.30
Mutated *FLT3* (Yes/ No)	0.815	1.143	0.375	3.482
Mutated *IDH1* (Yes/ No)	0.914	1.079	0.273	4.255
Mutated *IDH2* (Yes/ No)	0.488	0.631	0.172	2.317
Mutated *JAK2* (Yes/ No)	0.573	0.647	0.143	2.935
Mutated *KDM6A* (Yes/ No)	0.088	0.026	0	1.722
Mutated *KIT* (Yes/ No)	0.900	1.140	0.148	8.79
Mutated *KMT2A* (Yes/ No)	**0.006**	**6.684**	**1.732**	**25.79**
Mutated *KRAS* (Yes/ No)	0.561	1.911	0.215	16.97
Mutated *MPL* (Yes/ No)	0.890	1.273	0.042	38.97
Mutated *NF1* (Yes/ No)	**0.005**	**9.013**	**1.927**	**42.15**
Mutated *NPM1* by NGS (Yes/ No)	0.130	2.933	0.729	11.80
Mutated *NRAS* (Yes/ No)	**0.021**	**0.043**	**0.003**	**0.623**
Mutated *PHF6* (Yes/ No)	**0.014**	**7.935**	**1.514**	**41.59**
Mutated *PRPF40B* (Yes/ No)	0.523	2.582	0.141	47.33
Mutated *RAD21* (Yes/ No)	0.851	1.330	0.067	26.33
Mutated *RUNX1* (Yes/ No)	0.969	0.977	0.299	3.195
Mutated *SETBP1* (Yes/ No)	0.652	0.631	0.085	4.681
Mutated *SF3A1* (Yes/ No)	0.670	2.090	0.07	62.14
Mutated *SF3B1* (Yes/ No)	0.236	2.380	0.567	10
Mutated *SH2B3* (Yes/ No)	0.794	1.416	0.104	19.29
Mutated *SRSF2* (Yes/ No)	0.637	1.332	0.405	4.378
Mutated *STAG2* (Yes/ No)	0.640	1.480	0.286	7.664
Mutated *TET2* (Yes/ No)	0.944	1.036	0.383	2.807
Mutated *TP53* (Yes/ No)	**0.039**	**0.179**	**0.035**	**0.918**
Mutated *U2AF1* (Yes/ No)	**0.040**	**7.085**	**1.092**	**45.97**
Mutated *VHL* (Yes/ No)	0.134	10.09	0.492	206.8
Mutated *WT1* (Yes/ No)	0.175	0.189	0.017	2.095
Mutated *ZRSR2* (Yes/ No)	0.942	1.058	0.228	4.904
Score response predictor (no/ yes)	0.341	0.664	0.286	1.543
Score High Risk (no/yes)	0.814	1.200	0.263	5.468

Table S4. Logistic regression analysis. Response defined as overall response after 3rd cycle in the global series in function of characteristics of patients with AML on the treatment arm. Responder patients were defined as patients who achieved CR, CRi or PR (overall response) after 3rd cycle. AZA = azacitidine. FLUGA = fludarabine plus low-dose cytarabine (LDAC). OR = odds ratio. CI = confidence interval. Cytogenetic Risk Group (Low-Intermediate / High-Risk. classification ELN 2017). The multivariate analyses showed that no variable was associated with achieving a CR after bonferroni adjustment.

**Parameter**	***p* Value**	**OR**	**95% CI for OR**	
			**Lower**	**Upper**
Median age. years	0.078	0.938	0.873	1.007
Cytogenetic (Low-Intermediate/High Risk)	0.241	0.624	0.284	1.373
AZA vs FLUGA-arm	0.928	1.047	0.388	2.827
Mutated *ASXL1* (Yes/No)	0.639	0.765	0.25	2.339
Mutated *BCOR* (Yes/ No)	0.252	0.322	0.046	2.237
Mutated *BCORL1* (Yes/ No)	0.489	0.502	0.071	3.532
Mutated CALR (Yes/ No)	0.999	0	0	
Mutated *CBL* (Yes/ No)	0.859	1.262	0.098	16.24
Mutated *CEBPA* by NGS (Yes/ No)	0.206	0.33	0.059	1.843
Mutated *DNMT3A* (Yes/ No)	0.379	0.62	0.214	1.799
Mutated *EPAS1* (Yes/ No)	0.836	1.222	0.183	8.154
Mutated *EPOR* (Yes/ No)	0.468	3.391	0.125	92.04
Mutated *ETV6* (Yes/ No)	0.306	0.38	0.059	2.429
Mutated *EZH2* (Yes/ No)	0.732	1.363	0.232	7.988
Mutated *FLT3* (Yes/ No)	0.244	0.538	0.189	1.527
Mutated *IDH1* (Yes/ No)	0.323	0.526	0.147	1.881
Mutated *IDH2* (Yes/ No)	0.779	0.845	0.262	2.727
Mutated *JAK2* (Yes/ No)	0.12	0.313	0.072	1.354
Mutated *KDM6A* (Yes/ No)	0.206	0.109	0.004	3.367
Mutated *KIT* (Yes/ No)	0.182	3.633	0.547	24.14
Mutated *KMT2A* (Yes/ No)	**0.025**	**4.29**	**1.204**	**15.30**
Mutated *KRAS* (Yes/ No)	0.348	2.45	0.377	15.92
Mutated *MPL* (Yes/ No)	0.827	0.692	0.026	18.69
Mutated *NF1* (Yes/ No)	0.033	4.538	1.13	18.21
Mutated *NPM1* by NGS (Yes/ No)	0.589	1.421	0.397	5.091
Mutated *NRAS* (Yes/ No)	**0.031**	**0.136**	**0.022**	**0.832**
Mutated *PHF6* (Yes/ No)	0.102	3.767	0.767	18.50
Mutated *PRPF40B* (Yes/ No)	0.778	1.506	0.088	25.84
Mutated *RAD21* (Yes/ No)	0.642	1.954	0.116	32.95
Mutated *RUNX1* (Yes/ No)	0.929	1.051	0.35	3.159
Mutated *SETBP1* (Yes/ No)	0.681	0.692	0.12	4.001
Mutated *SF3A1* (Yes/ No)	0.399	4.261	0.147	123.6
Mutated *SF3B1* (Yes/ No)	0.275	2.106	0.553	8.019
Mutated *SH2B3* (Yes/ No)	0.705	1.614	0.136	19.18
Mutated *SRSF2* (Yes/ No)	0.348	1.673	0.571	4.904
Mutated *STAG2* (Yes/ No)	0.766	1.262	0.273	5.822
Mutated *TET2* (Yes/ No)	**0.070**	**2.347**	**0.934**	**5.9**
Mutated *TP53* (Yes/ No)	**0.018**	**0.190**	**0.048**	**0.756**
Mutated *U2AF1* (Yes/ No)	0.132	3.569	0.681	18.71
Mutated *VHL* (Yes/ No)	0.141	8.857	0.484	161.9
Mutated *WT1* (Yes/ No)	0.414	0.420	0.052	3.370
Mutated *ZRSR2* (Yes/ No)	0.172	2.587	0.660	10.13
Score response predictor (no/ yes)	0.471	0.704	0.271	1.828
Score High Risk (no/yes)	0.983	0.975	0.094	10.12

Table S5. Median OS in function mutant versus wild-type genes. Only genes mutated in ≥ 4% of patients were included in this table.

Gene	AZA-Arm			FLUGA-Arm		
	% Mutated Patients	wt Median OS Months (95% CI)	Mut Median OS Months (95% CI)	% Mutated Patients	wt Median OS Months (95% CI)	Mut Median OS Months (95% CI)
ASXL1	21.6	9 (2.3–15.7)	12 (0.4–23.6)	21.4	4(2.6–5.4)	7(0–14.8)
BCOR	4.1	11 (4.3–17.7)	7 (0–17.8)	7.1	5 (3.0–6.9)	3 (0–11.3)
BCORL1	4.1	11(5.0–16.9)	4 (NA)	6.3	5 (2.9–7.1)	4 (0–13.6)
CEBPA	6.2	11 (5.6–16.4)	3 (0–0.7)	5.4	5 (3.0–6.9)	3 (0–11.4)
DNMT3A	22.7	11 (4.4–17.5)	9 (2.5–15.5)	20.5	5 (2.4–7.6)	4 (2.0–5.9)
ETV6	7.2	11 (4.5–17.5)	9 (3.9–14.1)	5.4	5 (2.8–7.1)	4 (1.8–6.1)
EZH2	9.3	7 (0.7–13.3)	14 (6.5–21.5)	8.0	5 (2.7–7.3)	4 (0–8.6)
FLT3	25.8	11 (4.3–17.7)	10 (0–29.9)	24.1	5 (2.9–7.0)	3 (1.3–4.66)
IDH1	12.4	11 (4.8—17.2)	7 (0–20.4)	19.6	4 (2.5–5.5)	7 (2.4–11.6)
IDH2	16.5	10 (2.7–17.3)	12 (2.3–21.7)	17.9	5 (3.4–6.5)	6(2.1–9.9)
JAK2	8.2	11 (5.1–16.9)	1 (NA)	7.1	5 (2.9–7.1)	1 (0–6.5)
KIT	5.2	11 (4.5–17.5)	7 (0.6– 13.4)	5.4	5 (3.0–6.9)	3 (0–7.8)
KMT2A	11.3	9 (3.4–4.6)	17 (0–35.4)	7.1	5 (3.0–6.9)	3 (0–12.7)
KRAS	6.2	11 (5.1–16.9)	1 (0–5.8)	4.5	5 (3.0–6.9)	2 (0–4.1)
NF1	13.4	12 (5.7–18.3)	4 (0–11.9)	8.9	5 (2.9–7.1)	4 (0–11.7)
NPM1	15.5	9 (2.6–15.4)	16 (0.9–31.1)	16.1	5 (2.8–7.1)	5 (0–11.2)
NRAS	5.2	10 (4.4–15.6)	15 (0–38.6)	16.1	6 (3.8–8.1)	2 (0.6–3.4)
PHF6	6.2	10 (3.5–16.5)	12 (0–27.7)	5.4	5 (2.9–7.1)	4(0–13.6)
RUNX1	23.7	7 (0–14.0)	15 (7.7–22.2)	17.9	5 (3.0–6.9)	3 (1.3–4.7)
SETBP1	6.2	11 (5.1–16.8)	4 (NA)	2.7	5 (2.9–7.0)	Not reached
SF3B1	8.2	9 (2.8–15.2)	19 (9.6–28.4)	6.3	5 (3.0–6.9)	11 (0–34.1)
SH2B3	6.2	9 (1.5–16.5)	11 (8.8–13.1)	2.7	5 (2.8–7.1)	11 (1.4–20.6)
SRSF2	22.7	11 (4.4–17.6)	7 (0–20.3)	24.1	4 (2.8–5.2)	11 (3.6–18.4)
STAG2	8.2	10 (3.8–16.2)	11 (0–26.4)	10.7	5 (2.9–7.1)	3 (0.7–5.2)
TET2	23.7	7 (0.1–13.9)	14 (9.8–18.2)	28.6	5 (2.6–7.4)	4 (1.2–6.7)
TP53	22	14 (10.9–17.0)	2 (0.4–3.5)	19.6	7 (4.4–9.5)	2 (0.9–3.1)
U2AF1	8	7 (1.3–12.7)	15 (8.3–21.7)	7.1	5 (3.4–6.6)	6 (1.8–10.1)
WT1	8	11 (4.9–17.0)	4 (0–10.4)	7.1	5 (2.9–7.1)	3 (2.1–3.9)
ZRSR2	9	7 (1.1–12.9)	15 (8.5–21.5)	8.0	5 (3.5–6.5)	7 (0–18.7

OS = Overall Survival. Overall survival was calculated from diagnosis to the time of death from any cause. In the AZA-arm. the median OS was 10 months (range 4.4–15.6) for wt-*NRAS* versus 15 months (range 0–38.6) for mut-*NRAS*, whereas in the FLUGA-arm the median OS was 6 months (range 3.8–8.1) for wt-*NRAS* versus 2 months (range 0.6–3.4) for mut-*NRAS* ($p = 0.013$). In the AZA-arm. the median OS was 14 months (range 10.9–17) for wt-*TP53* versus 2 months (range 0.4–3.7) for mut-*TP53*, whereas in the FLUGA-arm the median OS was 7 months (range 4.4–9.6) for wt-*TP53* versus 2 months (range 0.9–3.1) for mut-TP53 ($p < 0.001$). In addition, patients with mutated *SF3B1* showed a trend for better prognosis in both arms. In the AZA-arm. the median OS was 9 months (range 2.8–15.2) for wt-*SF3B1* versus 19 months (range 9.6–28.4) for mut-*SF3B1* ($p = 0.081$), whereas in the FLUGA-arm the median OS was 5 months (range 3–6.9) for wt-*SF3B1* versus 11 months (range 0–34.1) for mut-*SF3B1* ($p = 0.083$).

Table S6. Mutated genes in FLUGAZA clinical trial (AZA versus FLUGA).

Gene	AZA-Arm (Cases)	AZA-Arm (%)	FLUGA-Arm (cases)	FLUGA-Arm (%)
ASXL1	21	21.6	24	21.4
BCOR	4	4.1	8	7.1
BCORL1	4	4.1	7	6.3
CALR	1	1	1	0.9
CBL	2	2.1	4	3.6
CEBPA	6	6.2	6	5.4
CSF3R	0	0	0	0
DNMT3A	22	22.7	23	20.5
EGLN1	0	0	0	0
EPAS1	3	3.1	7	6.3
EPOR	1	1	4	3.6
ETV6	7	7.2	6	5.4
EZH2	9	9.3	9	8
FLT3	25	25.8	27	24.1
IDH1	12	12.4	22	19.6
IDH2	16	16.5	20	17.9
JAK2	8	8.2	8	7.1
KDM6A	3	3.1	3	2.7
KIT	5	5.2	6	5.4
KMT2A	11	11.3	8	7.1
KRAS	6	6.2	5	4.5
MPL	2	2.1	1	0.9
NF1	13	13.4	10	8.9
NPM1	15	15.5	18	16.1
NRAS	5	5.2	18	16.1
PHF6	6	6.2	6	5.4
PRPF40B	1	1	4	3.6
RAD21	2	2.1	2	1.8
RUNX1	23	23.7	20	17.9
SETBP1	6	6.2	3	2.7
SF3A1	0	0	2	1.8
SF3B1	8	8.2	7	6.3
SH2B3	6	6.2	3	2.7
SMC1A	1	1	2	1.8
SRSF2	22	22.7	27	24.1
STAG2	8	8.2	12	10.7
TET2	23	23.7	32	28.6
THPO	1	1	0	0
TP53	23	22	22	19.6
U2AF1	7	8	8	7.1
VHL	2	2	2	1.8
WT1	3	8	8	7.1
ZRSR2	8	9	9	8

Distribution of gene mutations in both arms of the clinical trial (AZA = azacitidine. FLUGA = fludarabine plus low-dose cytarabine (LDAC)). Number of cases and % with mutations in each arm.

Table S7. Univariate Cox regression analysis for OS and RFS.

Variables	HR	Risk of death 95% CI for HR		*p*-value	HR	Risk of relapse 95% CI for HR		*p*-value
		Lower	Upper			Lower	Upper	
Age. years	1.03	0.997	1.064	0.070	0.99	0.954	1.03	0.663
Cytogenetic (Low-Intermediate / High Risk)	1.23	0.756	2.006	0.404	0.92	0.530	1.583	0.754
AZA vs FLUGA-arm	1.26	0.854	1.847	0.245	1.61	1.034	2.495	0.034
ASXL1	1.03	0.630	1.692	0.898	0.95	0.544	1.657	0.856
BCOR	1.30	0.609	2.769	0.497	**3.79**	**1.696**	**8.442**	**0.001**
BCORL1	1.06	0.462	2.405	0.898	0.96	0.362	2.527	0.928
CALR	0.51	0.042	6.142	0.595	12.9	2.014	82.967	0.006
CBL	2.75	0.743	10.2	0.129	0.23	0.022	2.453	0.226
CEBPA	1.95	0.884	4.29	0.097	2.12	0.871	5.161	0.097
DNMT3A	1.46	0.915	2.325	0.112	1.42	0.784	2.565	0.247
EPAS1	0.90	0.322	2.473	0.828	1.35	0.436	4.185	0.601
EPOR	2.37	0.690	8.16	0.170	0.23	0.035	1.477	0.121
ETV6	2.05	0.930	4.521	0.074	1.40	0.583	3.371	0.449
EZH2	0.69	0.338	1.37	0.281	0.75	0.356	1.579	0.448
FLT3	1.16	0.713	1.871	0.557	1.07	0.589	1.949	0.818
IDH1	1.21	0.675	2.151	0.526	1.57	0.783	3.148	0.203
IDH2	1.53	0.881	2.641	0.131	1.32	0.695	2.508	0.394
JAK2	2.32	1.037	5.17	0.040	1.90	0.770	4.708	0.163
KDM6A	2.60	0.854	7.903	0.092	0.76	0.150	3.873	0.744
KIT	0.73	0.283	1.904	0.525	1.62	0.632	4.147	0.314
KMT2A	0.65	0.332	1.267	0.205	1.09	0.527	2.258	0.813
KRAS	1.05	0.427	2.589	0.912	0.38	0.108	1.309	0.124
MPL	0.64	0.129	3.18	0.586	2.14	0.407	11.23	0.369
NF1	1.41	0.726	2.753	0.308	1.55	0.736	3.247	0.249
NPM1	0.81	0.411	1.602	0.547	0.49	0.219	1.11	0.087
NRAS	**2.09**	**1.191**	**3.652**	**0.010**	2.62	1.300	5.269	0.007
PHF6	0.58	0.246	1.365	0.212	1.35	0.5345	3.401	0.526
PRPF40B	1.45	0.360	5.858	0.598	0.45	0.047	4.391	0.496
RAD21	0.89	0.228	3.461	0.866	2.97	0.673	13.119	0.150
RUNX1	1.16	0.688	1.951	0.579	1.51	0.868	2.619	0.144
SETBP1	0.63	0.208	1.887	0.406	0.99	0.347	2.87	0.998
SF3A1	1.76	0.356	8.725	0.487	0.86	0.093	7.836	0.891
SF3B1	0.64	0.339	1.214	0.172	0.77	0.364	1.619	0.488
SH2B3	0.55	0.181	1.666	0.290	1.71	0.477	6.107	0.410
SMC1A	1.24	0.315	4.895	0.756	0.91	0.156	5.282	0.915
SRSF2	0.76	0.449	1.285	0.304	0.75	0.424	1.331	0.327
STAG2	1.41	0.735	2.721	0.299	1.32	0.644	2.703	0.448
TET2	1.29	0.838	1.98	0.246	1.68	1.020	2.757	0.041
TP53	**2.57**	**1.351**	**4.872**	**0.004**	1.90	0.822	4.374	0.133
U2AF1	0.47	0.206	1.059	0.068	0.48	0.190	1.217	0.122
VHL	0.35	0.042	2.892	0.329	2.62	0.659	10.411	0.171
WT1	1.13	0.472	2.688	0.787	2.72	0.944	7.844	0.063
ZRSR2	0.82	0.400	1.669	0.581	1.17	0.555	2.472	0.678

AZA = azacitidine. FLUGA = fludarabine plus low-dose cytarabine (LDAC). HR = hazard ratio. CI = confidence interval. Cytogenetic Risk Group (Low-Intermediate / High-Risk. classification ELN 2017).

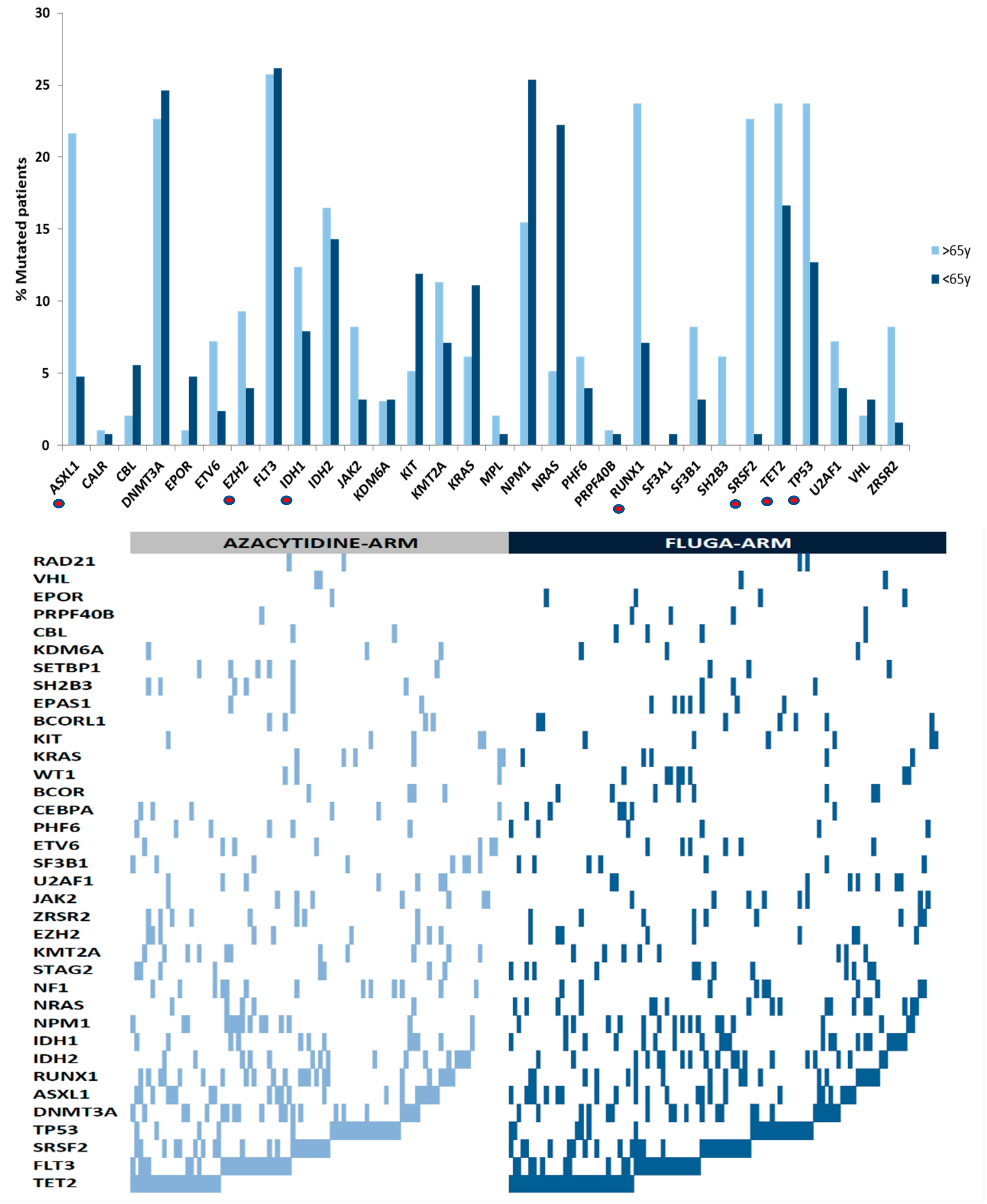

Figure S1. The landscape of mutated gene in elderly patients with AML is different to that of younger patients. (**A**). The mutational landscape described in the present study is different to that previously published for younger patients. We detected a higher number of patients with mutations in *ASXL1*, *EZH2*, *IDH1*, *RUNX1*, *SRSF2*, *TET2* and *TP53* in the elderly AML cohort versus our previously published younger AML cohort. (**B**). Distribution of mutations in both arms showing that *NRAS* mutations ($p = 0.012$) are more frequent in patients randomized to the FLUGA-arm. By contrast. *TP53* mutation frequency distribution is homogeneous: 23.7% in AZA-arm and 19.6% in FLUGA-arm (p = NS).

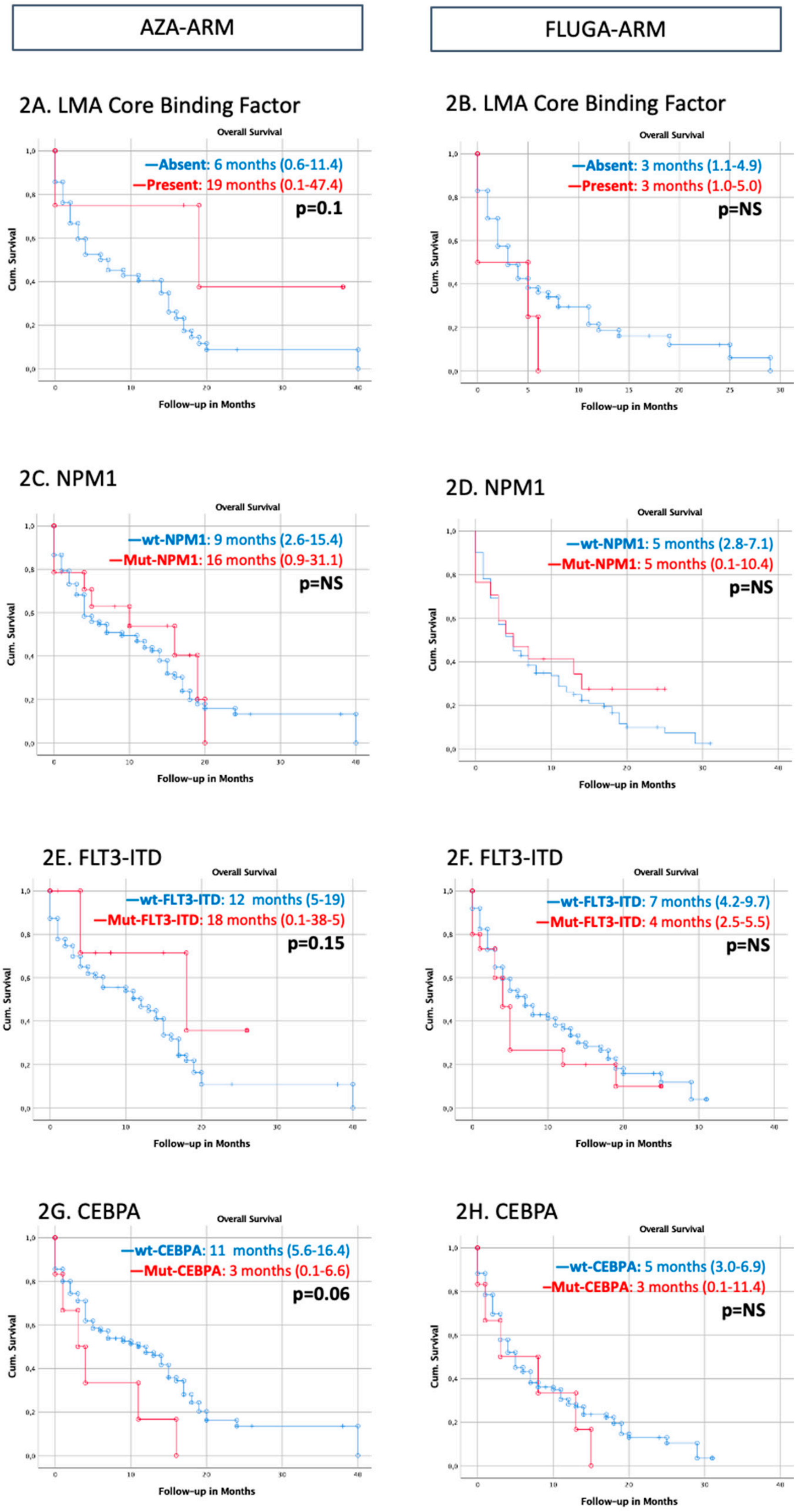

Figure 2. Kaplan-Meier analyses were performed to assess the association of conventional genomic aberrations with patient overall survival. No conventional molecular marker with prognostic impact on overall survival was detected**.** Recurrent genetic abnormalities group have no significant impact on overall survival (OS) in the AZA-arm (**A**) or the FLUGA-arm (**B**). *NPM1* or *FLT3*-ITD have no significant impact on OS in the AZA-arm (**C**,**E**. respectively) or the FLUGA-arm (**D**,**F**. respectively). *CEBPA* mutations showed a tendency for association with poor OS in the AZA-arm.

with a median OS of 11 months for *CEBPA* mutated (mut) versus 3 months for *CEBPA* wild type (wt); p = 0.06 (**G**,**H**. respectively). Absence of marker or wild-type status is indicated in blue. presence of marker or mutated status is represented in red. AZA. azacytidine; FLUGA fludarabine plus low-dose cytarabine (LDAC)

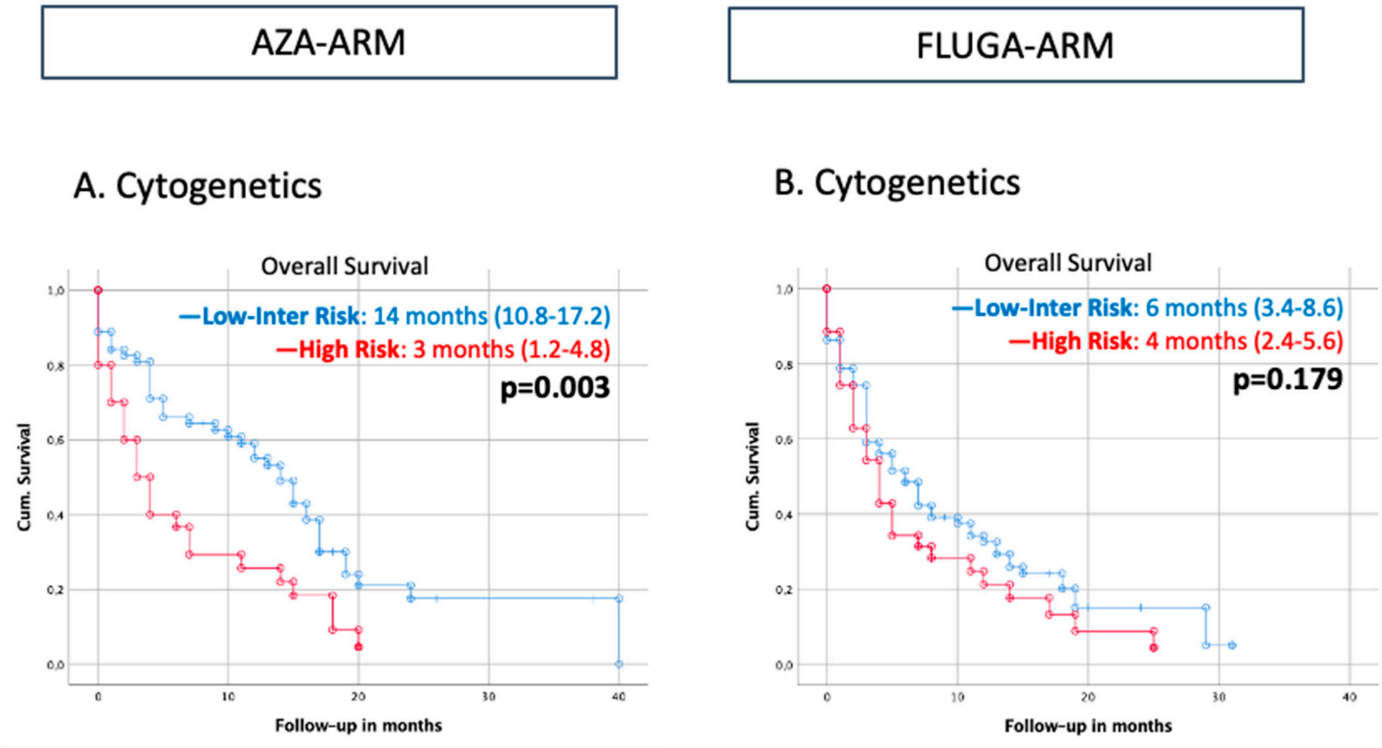

Figure S3. Kaplan-Meier analyses were performed to assess the association of conventional genomic aber-rations with patient overall survival. Cytogenetic risk predicts overall survival in elderly patients with AML in AZA-arm**.** Graphs represent survival of the low-intermediate-risk group (blue) versus high-risk group in AZA-arm (**A**) and in FLUGA-arm (**B**). Patients with low-intermediate-risk clearly gained benefit from AZA (median overall survival was 14 months in AZA-arm *vs* 6 months in FLUGA-arm; p = 0.003). AZA. azacytidine; FLUGA fludarabine plus low-dose cytarabine (LDAC).

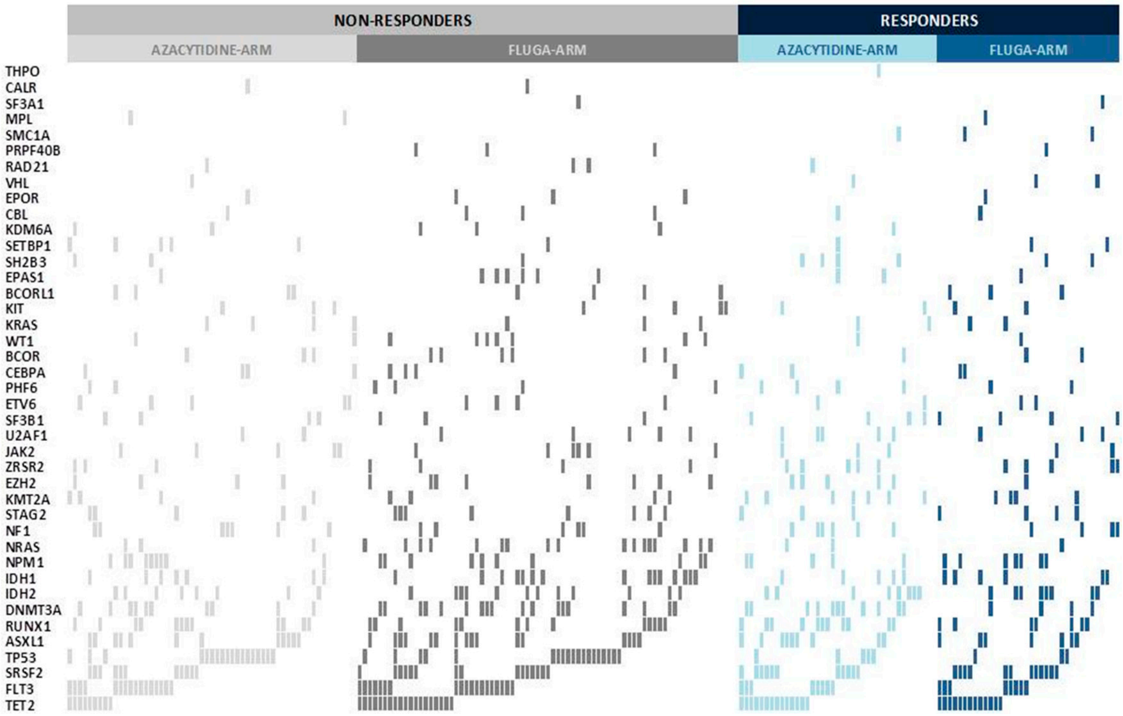

Figure S4. Mutational landscape in responders and non-responders in AZA-arm and FLUGA-arm after 3rd cycle. Each gene is represented by rows and the patients with mutations are shown in AZA-arm and FLUGA-arm and subdivided as responders and non-responders. Responders were defined as patients who achieve CR (complete remission) or CRi (complete remission with incomplete hematologic recovery). AZA = azacytidine; FLUGA = fludarabine plus low-dose cytarabine (LDAC).

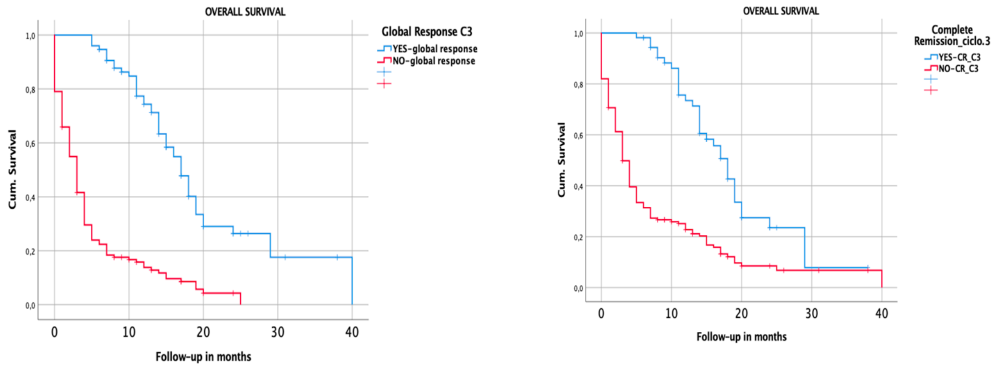

	Sig.	HR	95,0% CI for HR	
			Lower	Upper
Global Response Cycle 3	<0.001	5,005	2,707	9,254
Complete Remission Cycle 3	0,66	1,16	0,598	2,25

Figure S5. Achieving overall response after 3º cycle AZA or FLUGA is the main factor associated with increased Overall Survival.

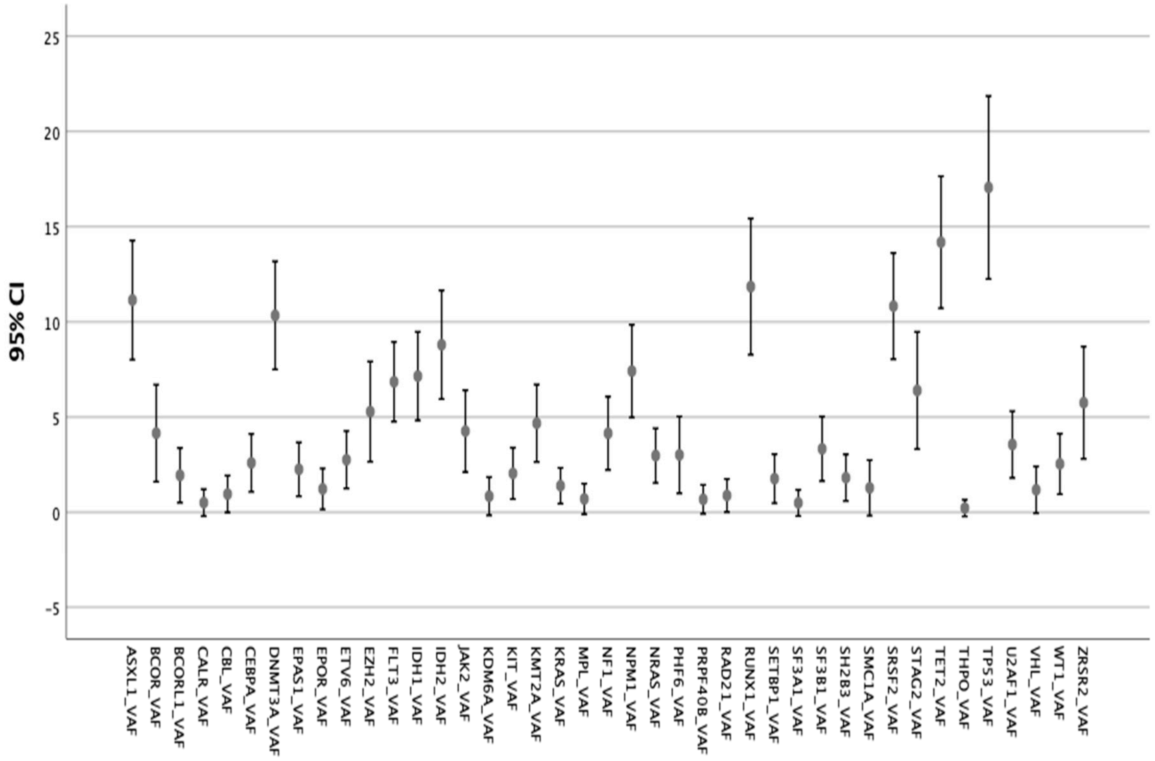

Figure S6. Variant allele frequency distribution by genes represented as median and 95% confidence interval. VAF (variant allele frequency) was defined as the percentage of sequence reads observed matching a specific DNA variant. divided by the overall coverage at that locus.

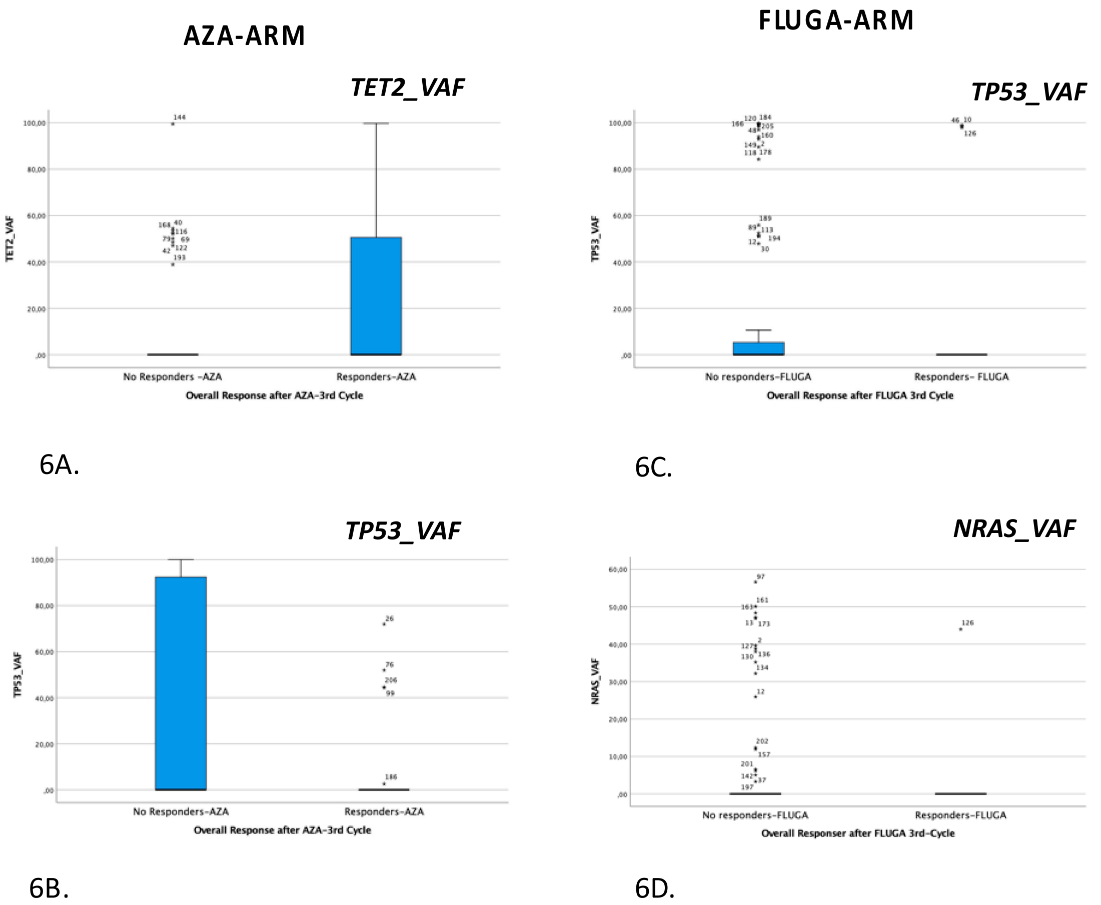

Figure S7. Differential distribution of variant allele frequency -gene mutations between responders and non-responders**.** VAF (variant allele frequency) was defined as the percentage of sequence reads observed matching a specific DNA variant. divided by the overall coverage at that locus. (**A**). Distribution of VAF-*TET2* in the AZA-arm is different between responders and non-responders: median 20.37 versus 8.7% (p = 0.022). (**B**). The distribution of VAF-*TP53* in the AZA-arm is different between responders and non-responders: median 5.52 versus 27.3% (p = 0.015). (**C**) The distribution of VAF-*NRAS* in the FLUGA-arm is different between responders and non-responders: median 1.22 versus 6.7% (p = 0.009). (**D**) The distribution of VAF-*TP53* in the FLUGA-arm is different between responders and non-responders: median 8.2 versus 19.64% (p = 0.046).
